# Hypoxia induces telomerase reverse transcriptase (TERT) gene expression in non-tumor fish tissues *in vivo*: the marine medaka (*Oryzias melastigma*) model

**DOI:** 10.1186/1471-2199-7-27

**Published:** 2006-09-11

**Authors:** Richard MK Yu, Eric XH Chen, Richard YC Kong, Patrick KS Ng, Helen OL Mok, Doris WT Au

**Affiliations:** 1 Department of Biology and Chemistry, City University of Hong Kong, 83 Tat Chee Avenue, Kowloon, Hong Kong

## Abstract

**Background:**

Current understanding on the relationships between hypoxia, hypoxia-inducible factor-1 (HIF-1) and telomerase reverse transcriptase (TERT) gene expression are largely based on *in vitro *studies in human cancer cells. Although several reports demonstrated HIF-1- mediated upregulation of the human *TERT *gene under hypoxia, conflicting findings have also been reported. Thus far, it remains uncertain whether these findings can be directly extrapolated to non-tumor tissues in other whole animal systems *in vivo*. While fish often encounter environmental hypoxia, the *in vivo *regulation of *TERT *by hypoxia in non-neoplastic tissues of fish remains virtually unknown.

**Results:**

The adult marine medaka (*Oryzias melastigma*) was employed as a model fish in this study. We have cloned and characterized a 3261-bp full-length TERT cDNA, *omTERT*, which encodes a protein of 1086 amino acids. It contains all of the functional motifs that are conserved in other vertebrate TERTs. Motif E is the most highly conserved showing 90.9–100% overall identity among the fish TERTs and 63.6% overall identity among vertebrates. Analysis of the 5'-flanking sequence of the *omTERT *gene identified two HRE (hypoxia-responsive element; nt. – 283 and – 892) cores. Overexpression of the HIF-1α induced *omTERT *promoter activity as demonstrated using transient transfection assays. The *omTERT *gene is ubiquitously expressed in fish under normoxia, albeit at varying levels, where highest expression was observed in gonads and the lowest in liver. *In vivo *expression of *omTERT *was significantly upregulated in testis and liver in response to hypoxia (at 96 h and 48 h, respectively), where concomitant induction of the *omHIF-1α *and erythropoietin (*omEpo*) genes was also observed. *In situ *hybridization analysis showed that hypoxic induction of *omTERT *mRNA was clearly evident in hepatocytes in the caudal region of liver and in spermatogonia-containing cysts in testis.

**Conclusion:**

This study demonstrates for the first time, hypoxic regulation of *TERT *expression *in vivo *in a whole fish system. Our findings support the notion that hypoxia upregulates *omTERT *expression via omHIF-1 in non-neoplastic fish liver and testis *in vivo*. Overall, the structure and regulation of the *TERT *gene is highly conserved in vertebrates from fish to human.

## Background

The enzyme telomerase, comprising a telomerase reverse transcriptase (TERT) subunit and a RNA subunit (TR), is ubiquitous from unicellular protozoa to mammals [1,2]. In humans, transcriptional regulation of *hTERT *is the major mechanism of telomerase activation. Hypoxia responsive elements (HREs), to which the hypoxia-inducible factor 1 (HIF-1) binds to regulate transcription of HIF-responsive genes, have been detected in the *hTERT *promoter [3,4]. Current understanding on the mechanistic relationship between hypoxia, hypoxia-inducible factor-1 (HIF-1) and *hTERT *gene expression is largely based on *in vitro *studies in human cancer cells. For instance, HIF-1 has been shown to transactivate *hTERT *expression in human choriocarcinoma cells [4], cervical cancer cells [3] and ovarian carcinoma cells [5]. Hypoxia induction of *hTERT *expression and subsequent telomerase activity have been reported for solid tumor cells (i.e. ovarian carcinoma and colon adenocarnoma cells) [6]. The HIF-1α protein has been shown to correlate well with *hTERT *expression in human placentas [4]. Conversely, Koshiji et al. [7] demonstrated that HIF-1α downregulates *hTERT *expression in human colon cancer cells, presumably via the displacement of Myc binding from the *hTERT *promoter. These conflicting findings suggest multiple mechanistic relationships between hypoxia, HIF-1α and *hTERT *expression in human cancer cells. Yet, it remains virtually unknown whether these findings can be directly extrapolated to non-tumor tissues in other whole animal systems *in vivo*.

Fish often encounter hypoxia in the aquatic environment, which is a pressing environmental problem worldwide [8-10]. Gracey et al. [11] used cDNA microarrays to study the hypoxia-induced gene expression profiles of the euryoxic fish, *Gillichthys mirabilis*, and found that genes encoding glycolytic enzymes and those involved in protein synthesis are downregulated, which support the notion that metabolic depression is an important strategy for survival under hypoxia [12-14]. Interestingly, induction of genes involved in the suppression of cell proliferation and growth has also been observed which could serve as bioindicators of lowered growth during hypoxia. For example, insulin-like growth factor binding protein-1 (IGFBP-1) which regulates the availability of insulin-like growth factors in the circulation is involved in growth regulation. The role of HIF-1 in modulation of the IGFBP-1 gene and its involvement in hypoxia-induced growth and developmental retardation have recently been reported in zebrafish [15,16]. Although a detailed pattern of response to hypoxia in fish is emerging, it is not clear at what level of hypoxia these responses are initiated and how the various responses are related to each other. So far, we know of no reports on the effect of HIF-1 on TERT transcription in any piscine system. Given the important roles that TERT plays in apoptosis, DNA repair, cell proliferation and growth [17-20], a better understanding of the relationship between hypoxia and fish *TERT *expression will shed light on the *in vivo *regulation of fish *TERT *and its role in adaptive and toxicological responses in fish to hypoxic stress.

To investigate hypoxic regulation of fish *TERT *expression *in vivo*, we isolated and cloned the TERT gene (*omTERT*) from the marine medaka, *Oryzias melastigma*, and examined its *in vivo *organ expression patterns in whole fish under normoxia and in response to hypoxic stress. Analysis of *omTERT *expression by quantitative RT-PCR and *in situ *hybridization showed significant *in vivo *induction of *omTERT *expression in fish livers and testes. Gene transactivation studies using luciferase reporter constructs showed that ectopic expression of omHIF-1α enhanced *omTERT *promoter activity *in vitro*. Taken together, our findings support the notion that hypoxia upregulates *omTERT *expression via omHIF-1 in non-tumor fish tissues *in vivo*.

## Results

### Isolation and characterization of the *omTERT *cDNA

Using degenerate RT-PCR and RACE, the full-length *omTERT *cDNA was isolated from *O. melastigma*. The nucleotide sequence has been deposited in GenBank under accession number DQ286654. The 3624-bp full-length cDNA contains an open reading frame (ORF) of 3261 bp, a 5'-untranslated region (UTR) of 117 bp, a 3'-UTR of 219 bp and a polyadenylated tail of 27 bp. A putative polyadenylation signal (AATAAA) is located 200 bp downstream of the stop codon (TAA) (data not shown). The open reading frame (ORF) of *omTERT *encodes a polypeptide of 1086 amino acids, with a molecular weight (MW) of 124.5 kDa and a p*I *of 9.60. The physical properties of omTERT are very similar to that of the Fugu TERT (ORF = 1074 a.a., MW = 122 kDa, p*I *= 9.65; [21]). Pairwise alignment showed that omTERT shares high sequence identity with the TERTs of orange-spotted grouper (63.1%; Au et al., unpublished observation) and Fugu [Genbank: AAX59693; 61.1%] and moderate sequence identity with the TERTs of mouse [Genbank: O70372; 34.8%], human [Genbank: NP_003210; 34.5%], frog [Genbank: AAG43537; 33.4%] and chicken [Genbank: AAS75793; 29.1%].

**Figure 1:**
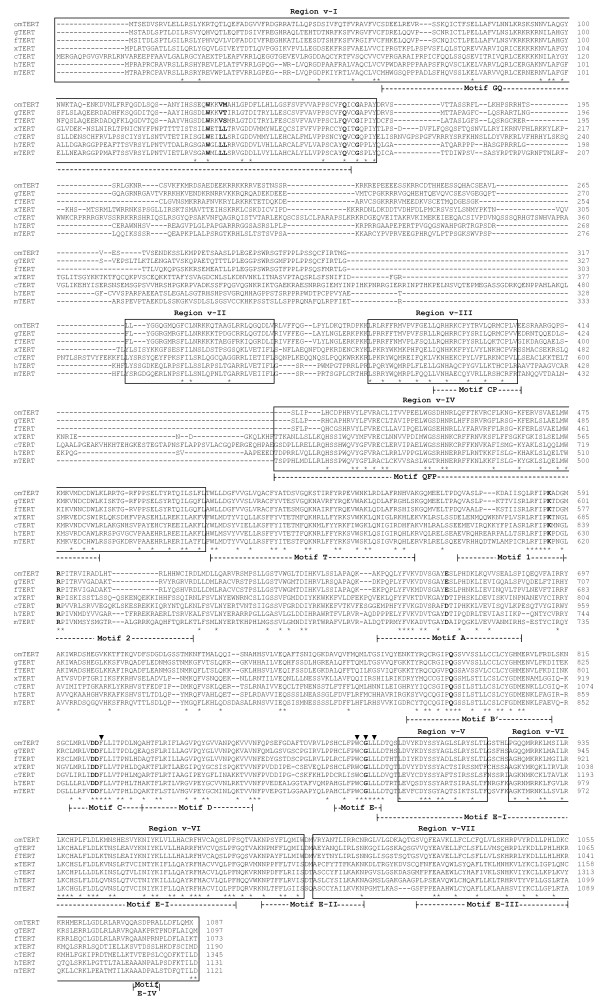
**Multiple alignment of the deduced amino acid sequence of *omTERT *with selected vertebrate TERT homologues**. TERT proteins from top to bottom are: omTERT (*O. melastigma*, this study), gTERT (orange-spotted grouper, *Epinephelus coioides*, unpublished, Genbank: DQ317442), fTERT (Fugu, Genbank: AAX59693), xTERT (*Xenopus laevis*, Genbank: AAG43537), cTERT (chicken, Genbank: AAS75793), hTERT (human, Genbank: NP_003210), mTERT (mouse, Genbank: NP_033380). Number on the right denotes the amino acid position. Identical amino acids shared by all protein sequences are marked with asterisks (*). Dashes (--) indicate gaps inserted for improved alignment. Residues known to be essential for telomerase activity are highlighted in bold. Conserved regions in the v-I to v-VII regions of vertebrate TERTs are labelled. Functional motifs: GQ, CP, QFP, T, 1, 2, A, B', C, D, E, E-I, E-II, E-III and E-IV are indicated by dashed lines and labelled underneath. Signature amino acid residues distinguishing TERT from other RTs are indicated by inverted triangles (▼).

Multiple sequence alignment indicated that omTERT contains all of the functional motifs that are highly conserved in TERTs from other vertebrate species (Fig. 1; [22-26]). Moreover, sequence comparison of homologus domains from different TERTs indicated that motifs C (58.8%) and E (63.6%) are most highly conserved (Table 1). These two motifs in omTERT contain signature residues that distinguish telomerase RT from other RTs – the aromatic residue following the two aspartic acid residues in motif C and the tryptophan-X-glycine-X-serine/leucine residues in motif E (Fig. 1; [27]). These signature sequences along with the TERT-specific T motif indicate that omTERT is a vertebrate TERT. Additionally, conserved amino acid residues known to be indispensable for telomerase activity [22,23,28] are also found in motifs GQ, 1, 2, B', C and E in omTERT (highlighted in bold in Fig. 1). Interestingly, the telomerase activity-determining aspartic acid in motif A of non-teleost TERTs is replaced by glutamic acid in all the fish TERTs. While negligible homology is present among the non-functional flexible linker region (the sequence stretch between regions v-I and v-II; [23]) in the TERTs examined, the length of this linker region in fish TERTs (*O. melastigma *contains 144 residues; grouper 153 residues; and Fugu 129 residues) are considerably shorter than those of other vertebrate TERTs (for example, frog contains 201 residues, chicken 301 residues, human 155 residues and mouse 160 residues).

### *In vivo *expression and response pattern of *omTERT *to hypoxia

**Figure 2:**
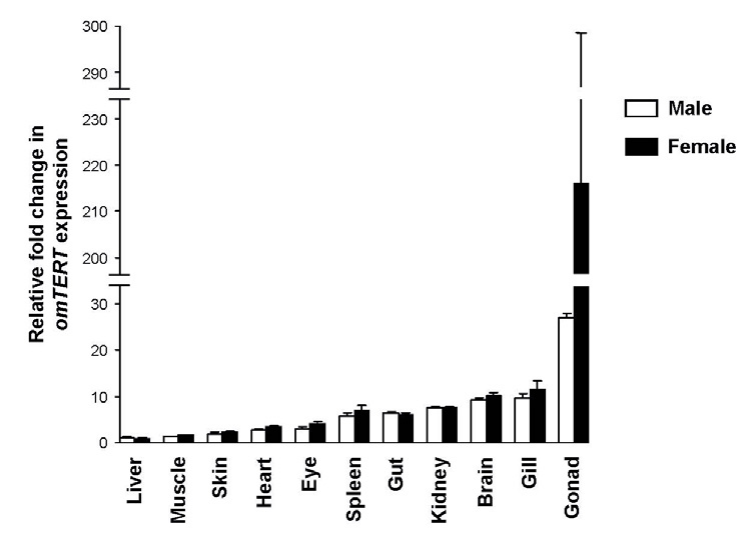
***In vivo *expression of *omTERT *mRNA**. Expression of *omTERT *in various *O. melastigma *tissues of male (open bar) and female (solid bar) fish (*n *= 5) were quantified using real-time RT-PCR and normalized against 18S rRNA. Data are presented as mean relative fold changes ± SEM with respect to expression level in the liver of female fish (arbitrarily set to 1).

Real-time PCR revealed ubiquitous expression of *omTERT *mRNA in all of the 11 organs examined in both male and female adult fish (Fig. 2), where highest expression was observed in the fish gonads. Moderate *omTERT *expression was detected in gill, brain, kidney, gut and spleen followed by eye, heart, skin, muscle. *omTERT *was expressed only very weakly in liver.

**Figure 3:**
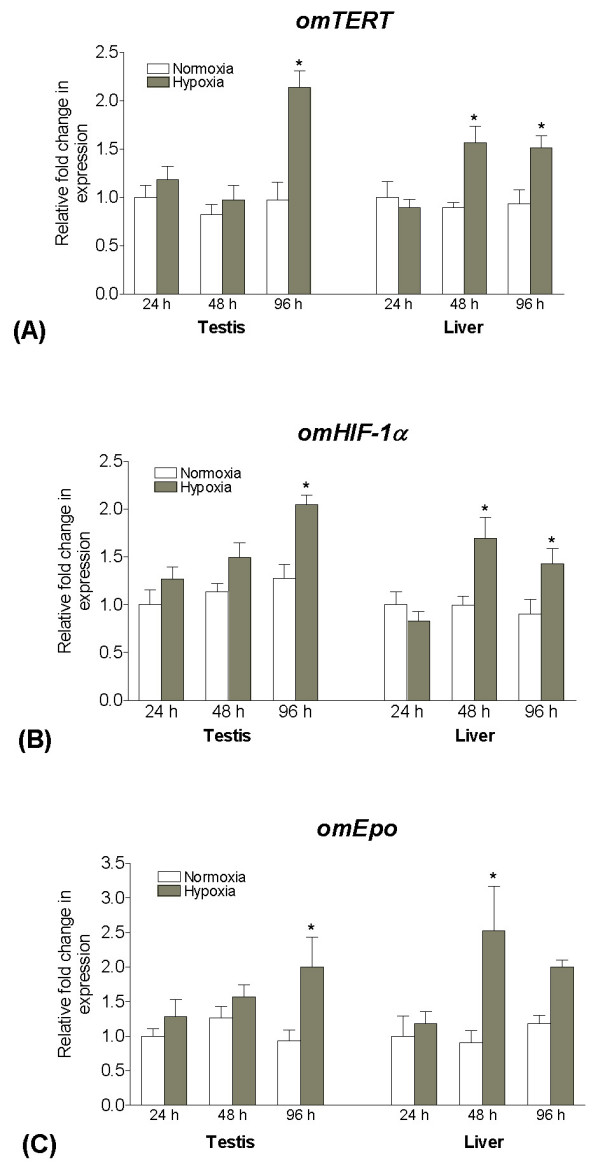
**Hypoxic induction of *omTERT*, *omHIF-1α *and *omEpo *expressions in liver and testis of marine medaka *in vivo*. **Male fish (*n *= 5) were exposed to either normoxic (6.4 mg O_2 _L^-1^) or hypoxic (1.8 mg O_2 _L^-1^) conditions for 24, 48 and 96 h. Expression of (A) *omTERT*, (B) *omHIF-1α *and (C) *omEpo *in the livers and testes was quantified using real-time RT-PCR and normalized with 18S rRNA. Data are presented as mean relative fold change ± SEM with respect to the gene expression level in the normoxic tissue sample at 24 h (arbitrarily set to 1). Asterisk (*) indicates significant difference from the other treatment group (*p *< 0.05).

We next compared the expression of *omTERT *mRNA under normoxia and hypoxia in two fish organs showing the highest (gonads) and lowest (liver) endogenous *omTERT *expression levels. Since a large variation in *omTERT *expression was found among different fish ovary samples, testes instead of ovaries were used. Upon exposure to hypoxia for 24 h and 48 h, no significant difference in expression of *omTERT *(Fig. 3A),* omHIF-1α *(Fig. 3B) and the HIF-regulated *omEpo *gene (Fig. 3C) was observed when compared to normoxic fish testes. Upon exposure to prolonged hypoxia for 96 h, *omTERT *in testes was induced by 2.2 ± 0.2 fold (*p *< 0.05; Fig. 3A) with a concomitant increase in *omHIF-1α *(1.6 ± 0.1 fold; *p *< 0.05; Fig. 3B) and *omEpo *(2.2 ± 0.5 fold; *p *< 0.05; Fig. 3C) expression. In liver, *omTERT *was hypoxically induced as early as 48 h (1.8 ± 0.2 fold) and the level was sustained until 96 h (1.6 ± 0.1 fold; *p *< 0.05; Fig. 3A). At these two time points, hypoxic induction of *omHIF-1α *(48 h: 1.7 ± 0.2 fold (*p *< 0.05); and 96 h: 1.6 ± 0.2 fold (*p *< 0.05); Fig. 3B) and *omEpo *(48 h: 2.8 ± 0.7 fold (*p *< 0.05); and 96 h: 1.7 ± 0.1 fold (p = 0.08); Fig. 3C) was also observed in the liver.

**Figure 4:**
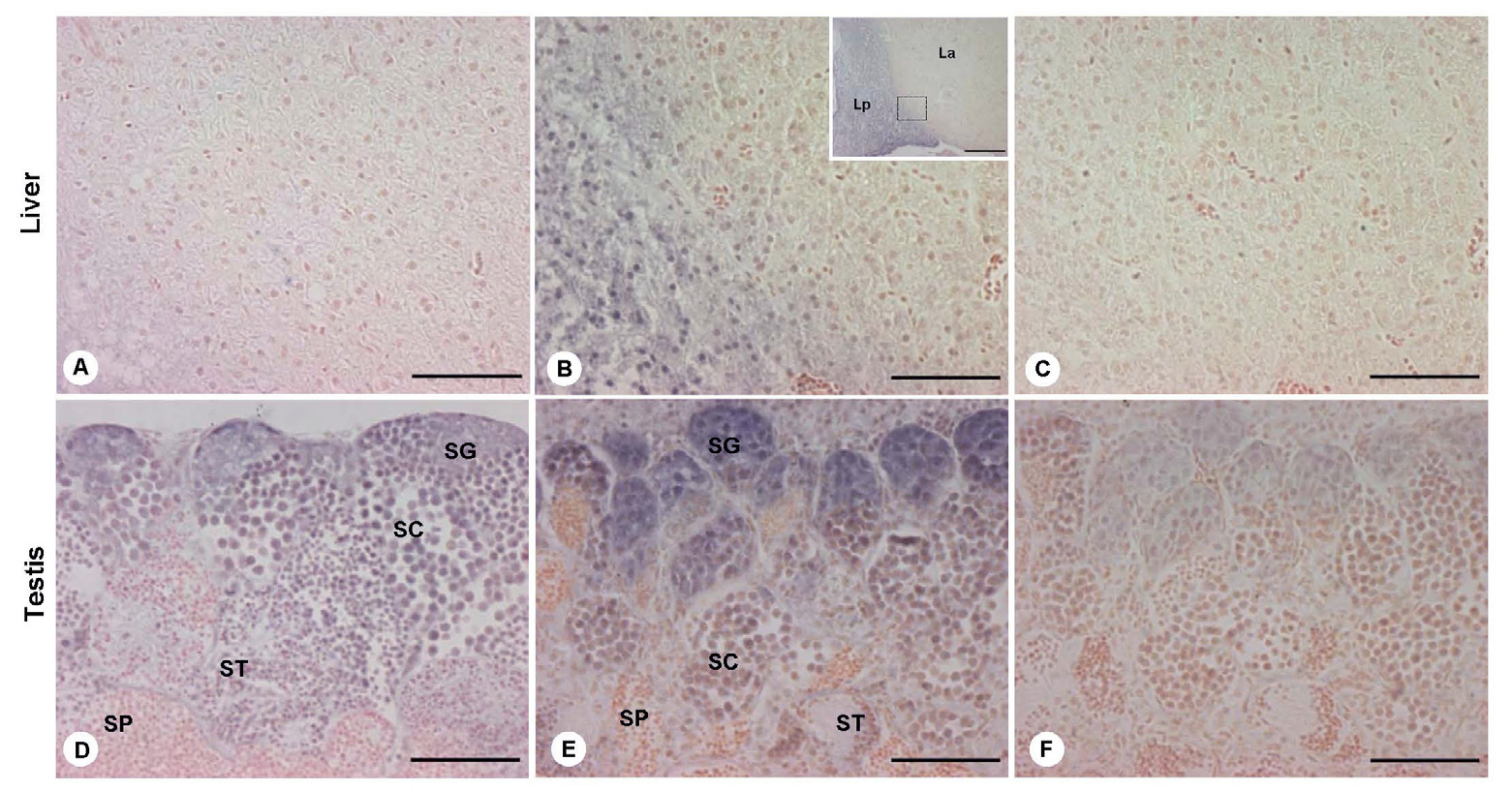
***In situ *localization of *omTERT *mRNA in livers (A-C) and testes (D-F) of marine medaka**. (A) Liver under normoxia (6.4 mg O_2 _L^-1^). Weak expression of *omTERT *mRNA (blue) is observed in liver parenchyma. (B) Liver exposed to hypoxia (1.8 mg O_2 _L^-1^) for 96 h. Induced expression of *omTERT *mRNA (blue) is evident, particularly at the posterior or caudal region of liver (Lp) as compared to the rostal or anterior region (La) (see insert at low magnification). (C) Adjacent liver section of (B) hybridized with an *omTERT *sense riboprobe as a negative control. (D) Testis under normoxia (6.4 mg O_2 _L^-1^). Expression of *omTERT *mRNA (blue) is strong in cysts containing spermatogonia (SG) and spermatocyte (SC), moderate in differentiating spermatids (ST) and absent in mature spermatozoa (SP) (red). (E) Testis exposed to hypoxia (1.8 mg O_2 _L^-1^) for 96 h. Induction of *omTERT *mRNA (blue) is conspicuous in spermatogonia (SG) and less prominent in other testicular cells. (F) Adjacent testis section of (E) hybridized with an *omTERT *sense riboprobe as a negative control. Scale bars = 50 μm, except insert of (b) = 200 μm.

*In situ *hybridization analysis showed that under normoxia, *omTERT *mRNA was only weakly expressed in the liver parenchyma (Fig. 4A). At 96 h hypoxia, *omTERT *expression was induced significantly in the caudal region of liver (Fig. 4B). In testis, *omTERT *expression was localized mainly in cysts containing highly proliferative sperm cells (i.e. spermatogonia and spermatocytes) and differentiating spermatids but not in mature spermatozoa (Fig. 4D). Upon exposure to hypoxia for 96 h, cysts containing spermatogonia showed the strongest induction of *omTERT *expression (Fig. 4E).

### *omTERT *5'-flanking sequence

**Figure 5:**
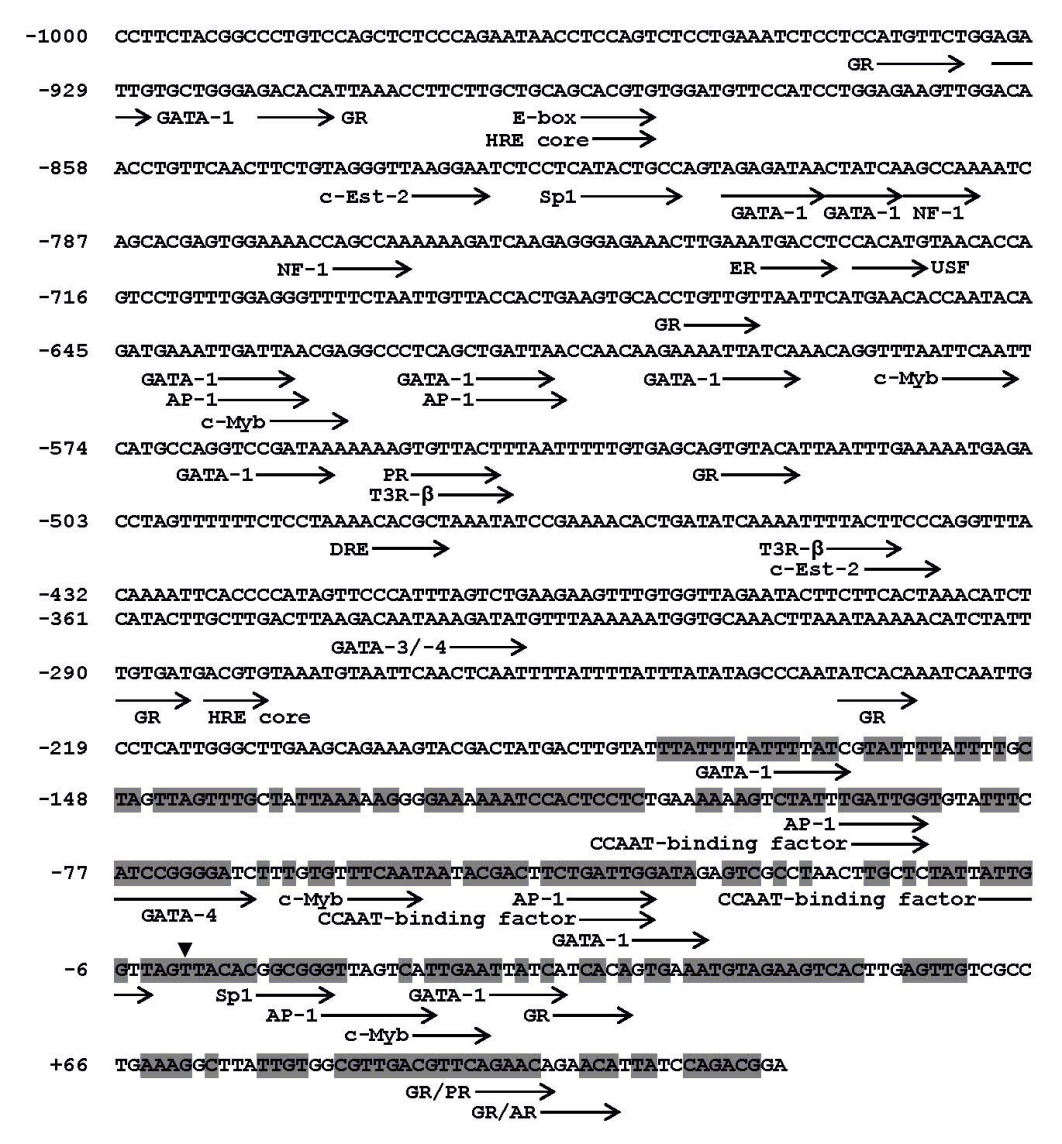
**The 5'-flanking sequence of *omTERT***. Numbers on the left denote the nucleotide positions relative to the transcription start site (designated as +1 and indicated by an inverted triangle). Positions of putative transcription factor binding sites are marked with arrows underneath. The 177-bp sequence in the *omTERT *promoter which is conserved also in the 5'-flanking sequence of the *O. latipes TERT *promoter is shaded in gray.

PCR amplification of a *Stu*I Genome Walking library (of *O. melastigma*) yielded a 3.6-kb genomic fragment which contains 3440-bp of 5'-flanking sequence and 185-bp 5'-UTR of the *omTERT *gene. The sequence has been deposited in GenBank under accession number DQ286655. Similar to the *TERT *promoters of Fugu, chicken and human [21,26,29], the *omTERT *5'-flanking sequence lacks a canonical TATA box but contains three putative CCAAT boxes near the transcription start site (TSS; at nt positions – 10, – 41 and – 91; Fig. 5). In contrast to *hTERT *and *cTERT*, no CpG island is found in the *omTERT *promoter, which is notably AT-rich (60%). Comparison of the *omTERT *and *fTERT *5'-flanking sequences using the PromoterWise program (EMBL-EBI) [30] failed to detect any homologous region of significant sequence identity. Using BLAST, the *O. latipes TERT *(*olTERT*) promoter sequence was identified in the medaka genome database [31] (scaffold 3719 at the NIG DNA Sequencing Center, Japan), and a 177-bp region that is conserved in both the *omTERT *and the *O. latipes TERT *(*olTERT*) promoters (ca. 75% identity) upstream of the TSS was identified. Significant sequence homology (69%) was also identified in the 5'-UTRs of the *omTERT *and *olTERT *genes.

To identify potential transcription factor binding sites (TFBSs) in the *omTERT *promoter, the first 1000-bp sequence upstream of the TSS was analyzed using a filter string-based search supported by the TESS program [32]. Two putative HREs (hypoxia-responsive elements; at nt. – 283 and – 892) were identified which contain the exact HRE sequence (ACGTG) as the functional HRE described in the zfIGFBP-1 gene promoter of zebrafish [16]. However, a HIF-1 ancillary sequence (HAS) adjacent to the HRE, that affects hypoxia and HIF-1 responsiveness of the zfIGFBP-1 gene, is not present in the *omTERT *promoter. Moreover, a single putative HRE core (GCGTG) was also identified in the Fugu *TERT *promoter [Genbank: AY861384].

Other putative TFBSs of particular interest were also found: AP1 (nt. +11, -42, -92, -617 and -637), SP1 (nt. +6 and -822), NF-1 (nt. -770 and -797), E-box (nt. -893), GATA-1 (nt. +24, -168, -563, -598, -617, -637, -803, -811 and -932), GATA-3 (nt. -335), GATA-4 (nt. -77 and -335), USF (upstream stimulating factor; nt. -730), c-Myb (nt. +18, -59, -581 and -633), GR (glucocorticoid receptor; nt. +29, +94, +99, -234, -672, -918 and -941), PR (progesterone receptor; nt. +94 and -551), AR (androgen receptor; nt. -19), T3R-β (thyroid hormone receptor-β; nt. -448 and -549), ER (estrogen receptor; nt. -737) and c-Est-2 (nt. -445 and -835) and DRE (dioxin-responsive element; nt. -483). Of these putative TFBSs: AP1, SP1, USF, E-box, and ER are implicated in the regulation of *hTERT *transcription [4,33,34].

### Induction of *omTERT *promoter by omHIF-1α

**Figure 6:**
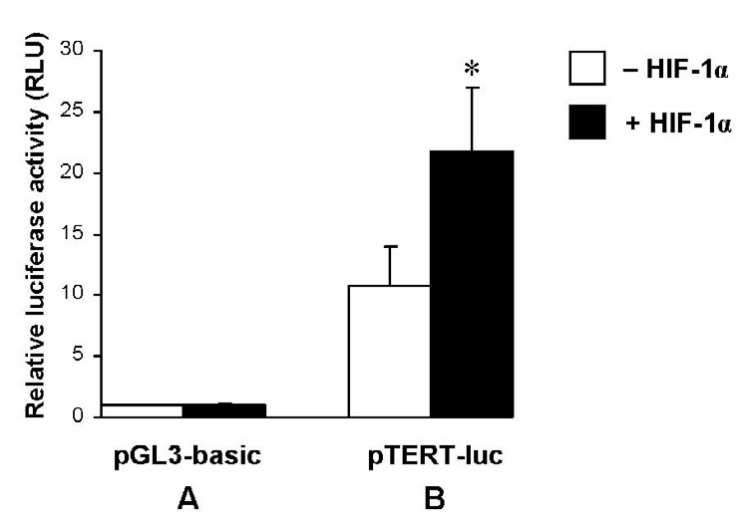
**Effect of omHIF-1α overexpression on *omTERT *promoter activity**. CHO Ka13 cells were transiently transfected with 600 ng of a luciferase reporter vector containing 3.5 kb of *omTERT *5'-flanking sequence (pTERT-luc; panel B) or 600 ng of empty reporter vector (pGL3-basic; panel A) along with 100 ng of pCMV-omHIF-1α (solid bar) or 100 ng of the empty pCMVTNT vector (open bar) and 100 ng of the pTracer-EF/Bsd/*lacZ *(β-galactosidase) expression vector. Relative luciferase activity (RLU) is the ratio of luciferase over β-galactosidase activity. Data are the mean ± SD of three independent experiments. Asterisk (*) indicates significant effect (*p *< 0.001) of omHIF-1α overexpression on induction of pTERT-luc activity.

To test the hypothesis that HIF-1α regulates *omTERT *transcription, the effect of omHIF-1α overexpression on luciferase activity driven by 3.5kb of *omTERT *promoter sequence was evaluated in HIF-1α-deficient CHO Ka13 cells. When cells were co-transfected with both pCMV-omHIF-1α and pTERT-luc, luciferase activity was significantly increased by ca. 2-fold when compared with cells transfected with pTERT-luc alone (*p *< 0.05) (Fig. 6).

## Discussion

The marine medaka, *O. melastigma*, is a hypoxia-tolerant species and can survive in a hypoxic environment (containing 0.8–1.8 mg O_2_/l) for 1–12 weeks with <10 % mortality (Au et al., unpublished observation). The hypoxia tolerance of *O. melastigma *was found to be comparable to that of other hypoxia-tolerant fish species such as the mudsucker, *Gillichthys mirabilis *[11] and the common carp, *Cyprinus carpio *[35]. In this study, we have cloned and characterized the expression pattern of the full-length telomerase reverse transcriptase (*omTERT*) cDNA from the marine medaka, *O. melastigma*. The deduced omTERT protein contains all of the functional motifs that are conserved in other vertebrate TERTs (Table 1), and shares particularly high sequence identity with the TERT of Fugu (61%) and moderate sequence identity (29–35%) with TERTs from other vertebrate species. Among the different motifs in TERT, motif E is the most highly conserved showing 90.9–100% overall identity among the fish TERTs and 63.6% overall identity among vertebrates (Table 1). Banik et al. [25] showed that the sequence spanning motif E to motif E-I is required for telomerase activity and curiously, analysis of the DNA sequences of the fish TERTs in this study showed that the lengths of motifs 1 and 2 (within the RT domain) are shorter by 3 and 8–11 residues, respectively, than other vertebrate TERTs. However, it is unclear what effect these changes may have on the RT activity of TERT. Overall, the findings of this study indicate that the structure and regulation of TERT is highly conserved in vertebrates from fish to human.

Real-time PCR analysis showed that *omTERT *is expressed ubiquitously, albeit at varying levels, in different organs of the adult marine medaka (Fig. 2). In particular, major expression of *omTERT *was detected in ovary and testis where germ cells are actively dividing and differentiating. *In situ *hybridization analysis of testes demonstrated that high *omTERT *expression is restricted to highly proliferating sperm cells (i.e. spermatogonia and spermatocytes) and differentiating spermatids but not in mature spermatozoa (Fig. 4D). This is in agreement with the findings of Achi et al. [36], which indicated a progressive decline in telomerase activity in the rat testes during sperm cell development. In this context, it was not surprising that *omTERT *was also found to be highly expressed in regenerative tissues such as gill, gut and kidney. A similarly high level of *omTERT *expression that was observed in the medaka brain (Fig. 2) may be attributed to the presence of undifferentiated neuronal precursor cells as was demonstrated in the *Xenopus *brain [37]. Recent evidence indicate that *TERT *expression in the brain enhances cell survival of developing neurons [17,18]. In this study, a low level of *omTERT *expression was also detected in liver and muscle tissues of the marine medaka. This is in agreement with the presence of telomerase activity in the muscle and liver tissues of adult zebrafish [38], rainbow trout [39] and marine medaka (Au et al., unpublished data).

Marine medaka exposed to prolonged hypoxia showed significant induction of *omTERT *expression in the testis (96 h) and liver (48 h), as determined by both quantitative RT-PCR and *in situ *hybridization techniques, that positively correlated with elevated expression levels of the *omHIF-1α *and *omEpo *genes (*p *< 0.05; Figs. 3B and 3C, respectively). Because of the unavailability of omHIF-1α-specific antibodies, detection of omHIF-1α protein by either Western blotting or immunohistochemistry was not possible, and expression of *omHIF-1α *and the HIF-1-regulated *omEpo *gene was therefore used as an indicator of hypoxic induction of omHIF-1 in this study. To determine whether *omTERT *is responsive to the HIF transcription factor, two putative HRE core sequences (ACGTG) were identified in the *omTERT *promoter by computer analysis (Fig. 5), and gene transfection experiments in CHO cells indicated that the *omTERT *promoter is upregulated by omHIF-1α (Fig. 6). The results strongly suggest that omHIF-1α has a role in the regulation of *omTERT *expression. And based on the molecular responses of *omTERT *to hypoxia in the medaka liver and testis (Figs. 3 and 4), it is highly likely that upregulated expression of *omTERT *under hypoxia is mediated (at least in part) via omHIF-1 in whole fish *in vivo*. The mechanistic relationships between omHIF-1α and omTERT (expression at the mRNA and protein levels), and the associated changes in omTERT telomerase activity in various fish organs under normoxia and hypoxia (and upon normoxic recovery) are currently being investigated in our laboratory to better understand the role(s) of these molecules in cell proliferation and growth.

The differential responses of hepatocytes (in caudal region of the liver) and testicular cells (in spermatogonia) in the marine medaka to hypoxic stress indicate that hypoxic induction of *TERT *is cell-type specific. Anti-apoptotic and protective functions of TERT against hypoxia-induced apoptosis and DNA damage have been demonstrated in a variety of mammalian cells (see review by Chung et al. [20]). It is tempting therefore to speculate that the increase in *omTERT *expression in medaka liver and testis under hypoxia could be a protective response to enhance survival of specific cell types. For instance, the role of TERT in maintaining the genomic stability of male germ cells is particularly critical during spermatogenesis to ensure the transfer of intact chromosomes to the offspring [36]. Additionally, the presence of a putative E-box (to which the c-Myc/Max heterodimer could potentially bind) and c-Myb binding sites in the *omTERT *promoter suggests that *omTERT *activation may aid in promoting cell proliferation and differentiation [40,41] by counteracting the potentially adverse effects of hypoxia on fish tissues. The molecular mechanisms operating through TERT in regulating cellular homeostasis and gametogenesis in fish certainly warrant further investigation. Functional studies and exposure experiments are currently underway in our laboratory to investigate the significance of the estrogen- (EREs) and dioxin-responsive (DREs) elements in the *omTERT *promoter on *in vivo *gene expression in the marine medaka. Conceivably, if *omTERT *expression is responsive to challenges by various estrogenic and/or dioxin-related pollutants, and the responses could be linked to Darwinian fitness traits (such as cell proliferation, growth and reproduction) in the marine medaka, this will open the possibility of TERT being used as a novel and meaningful molecular biomarker of effects for monitoring environmental stress and pollution in the marine environment [42].

## Conclusion

We report here the isolation and characterization of the *omTERT *gene from *O. melastigma*, a potential marine model fish for molecular ecotoxicology. Our findings suggest that hypoxic induction of *omTERT *expression in non-tumor liver and testis in whole fish system may be mediated, in part via the omHIF-1 transcription factor. Data from the marine medaka model suggest that the structure and regulation of TERT gene is conserved in vertebrates from fish to human.

## Methods

### Marine medaka

The marine medaka, *Oryzias melastigma*, can complete its entire life cycle in full strength seawater. Ubiquitous somatic telomerase activity was detected in *O. melastigma *which also exhibits telomere lengths (6–12 kb) resembling those of human tissues (8–15 kb) (Au et al., unpublished data). *O. melastigma *were purchased from a commercial hatchery in Taiwan and were maintained in 30 ‰ artificial seawater at 5.8 ± 0.2 mg O_2 _L^-1^, 28 ± 2°C in a 14 h light/10 h dark cycle. For hypoxia exposure experiments, adult fish were used and divided into two groups. The first group was maintained in a hypoxic system at (1.8 ± 0.2 mg O_2 _L^-1^) and the second group in a normoxic system (5.8 ± 0.2 mg O_2 _L^-1^) for 24, 48 and 96 h. Dissolved oxygen was monitored continuously using dissolved oxygen meters and polarographic probes (Cole-Parmer 5643–00, Illinois, USA). After the exposure period, adult fish (*n *= 5 for each sex) were sampled and tissues were immediately dissected out, snap-frozen in liquid nitrogen and stored at -80°C until ready to be processed.

### RNA extraction and cloning of *omTERT *cDNA by degenerate RT-PCR

Total RNA was extracted from fish tissues using the TRIZOL reagent (Invitrogen) according to the manufacturer's instructions. First-strand cDNA was synthesized using 1 μg total RNA, 1.25 μL dNTP (10 mM), 2.4 μL random hexamer (50 ng/μL), 1 μL RNaseOUT (40 U; Invitrogen) and 1 μL M-MLVRT (H-) (200 U/μL; Promega) in a total volume of 25 μL in 1 × M-MLVRT reaction buffer at 42°C for 50 min. The reaction was terminated by incubation at 70°C for 15 min. Using degenerate primers, fTERT-F (5'-TGGCTGAGCTGATGTGGAA-3'; sense) and fTERT-R (5'-TCATAGGCYCCACTCACRTC-3'; antisense), that target two regions highly conserved in the TERT sequences of Fugu (clone S001997 [43]) and rainbow trout (Genbank: CA380121, BX088059), RT-PCR was performed on first-strand cDNAs derived from total RNA of fish intestines. The RT-PCR mixture in 50 μL consisted of 3 μL of first-strand cDNAs (derived from fish intestinal total RNA), 1 μL of each degenerate primer (10 μM), 1 μL dNTP (10 mM), 5 μL 10 × PCR Buffer and 1 μL 50 × BD Advantage 2 polymerase mix (BD Biosciences). The PCR profile included heat denaturation at 95°C for 2 min followed by 35 cycles of heat denaturation at 95°C for 15 s, annealing at 55°C for 30 s and extension at 72°C for 1 min.

### Rapid amplification of cDNA ends (RACE)

The full-length *omTERT *cDNA was obtained from poly(A^+^)-selected RNA of whole fish by 5'- and 3'-RACE PCR using the Marathon cDNA Amplification kit (BD Biosciences). Nested PCR was performed using adaptor-specific primers, AP1 (5'-ATCAGGTCAGACAGCTGTGGGA-3'; outer) and AP2 (5'-ACTCACTATAGGGCTCGAGCGGC-3'; inner), along with two gene-specific primers (GSPs). The antisense GSPs for 5'-RACE were omTERT-5RACE-R1 (5'-aggagggacggcatggagcgcacac-3'; outer) and omTERT-5RACE-R2 (5'-gtctgccctgatgacccgagtgatgg-3'; inner) whereas the sense GSPs for 3'-RACE were omTERT-3RACE-F1 (5'-ggctttgttgtgggcctggtccagg-3', outer) and omTERT-3RACE-F2 (5'-gatcaggtggcgtccctccccaaagac-3', inner). A 50-μL PCR reaction contained 5 μL 10 × PCR buffer, 1 μL dNTP (10 mM), 1 μL GSP (10 μM), 1 μL adaptor primer (10 μM), 5 μL 1:50 diluted adaptor-ligated cDNA library (for primary PCR) or 1:50 diluted primary PCR product (for secondary PCR) and 1 μL 50 × BD Advantage 2 polymerase mix (BD Biosciences). The amplification profile for both primary and secondary PCRs consisted of 5 cycles of 94°C for 15 s and 72°C for 3 min, 5 cycles of 94°C for 15 s and 70°C for 3 min, and 25 cycles of 94°C for 15 s and 68°C for 3 min. Amplification products were purified and cloned into the pGEMT-Easy vector (Promega) for DNA sequencing. The full-length *omTERT *cDNA was obtained by full-length RT-PCR with primers targeting the cDNA ends using the PfuUltra DNA Polymerase (Stratagene).

### Real-time PCR

Real-time PCR was performed on the iCycler iQ Real-time PCR System (BioRad) using the SYBR Green I dye-based detection method. Similar protocols were also employed to quantify mRNA expression for two hypoxia marker genes, *omHIF-1α *and the erythropoietin gene (*omEpo*). First-strand cDNA was diluted to 1/10 and 5 μL were used for each real-time PCR reaction. Triplicates were run for PCR reaction. To normalize the target gene expression for differences in cDNA input, cDNA was diluted 1:2500 for measuring 18S rRNA levels. Diluted cDNAs (5 μL aliquots) were added to the wells of a 96-well thin-wall PCR plate, and to each was added 20 μL PCR master mix containing 12.5 μL 2 × iQ SYBR Green Supermix (BioRad), 0.5 μL of each target gene primer (10 μM) and 6.5 μL water or 1 μL of each 18S rRNA primer (10 μM) and 5.5 μL water. PCR primers were designed using the Primer3 program [44] and their sequences are shown in Table 2. The PCR plate was heated at 95°C for 2 min followed by 40 cycles of 95°C for 20 s, 60°C for 30 s and 72°C for 30 s. For quantification of PCR results, C_T _(the cycle at which the fluorescence signal is significantly different from background) was determined for each reaction. From a standard curve showing the relationship between the quantity of starting material (a dilution series of fish testis cDNA) and C_T_, PCR efficiency of the *omTERT *expression assay was determined to be 98.6% (R^2 ^= 0.999). Melting-curve analysis was performed by running a heat-dissociation protocol after the PCR to differentiate between the desired target amplicons and any primer-dimers or DNA contaminants. The size and identity of each gene amplicon were verified by agarose gel-electrophoresis and DNA sequencing, respectively. The C_T _values for both reference and target genes, and the efficiencies (E = 10-^1/*slope*^) of both assays were substituted into Equation (1) to yield the mean normalized expression (MNE) value.

MNE=(Ereference)CT reference, mean(Etarget)CT target, mean     (1)
 MathType@MTEF@5@5@+=feaafiart1ev1aaatCvAUfKttLearuWrP9MDH5MBPbIqV92AaeXatLxBI9gBaebbnrfifHhDYfgasaacH8akY=wiFfYdH8Gipec8Eeeu0xXdbba9frFj0=OqFfea0dXdd9vqai=hGuQ8kuc9pgc9s8qqaq=dirpe0xb9q8qiLsFr0=vr0=vr0dc8meaabaqaciaacaGaaeqabaqabeGadaaakeaacqqGnbqtcqqGobGtcqqGfbqrcqGH9aqpdaWcaaqaaiabcIcaOiabbweafnaaBaaaleaacqqGYbGCcqqGLbqzcqqGMbGzcqqGLbqzcqqGYbGCcqqGLbqzcqqGUbGBcqqGJbWycqqGLbqzaeqaaOGaeiykaKYaaWbaaSqabeaacqqGdbWqdaWgaaadbaGaeeivaqLaeeiiaaIaeeOCaiNaeeyzauMaeeOzayMaeeyzauMaeeOCaiNaeeyzauMaeeOBa4Maee4yamMaeeyzauMaeeilaWIaeeiiaaIaeeyBa0MaeeyzauMaeeyyaeMaeeOBa4gabeaaaaaakeaacqGGOaakcqqGfbqrdaWgaaWcbaGaeeiDaqNaeeyyaeMaeeOCaiNaee4zaCMaeeyzauMaeeiDaqhabeaakiabcMcaPmaaCaaaleqabaGaee4qam0aaSbaaWqaaiabbsfaujabbccaGiabbsha0jabbggaHjabbkhaYjabbEgaNjabbwgaLjabbsha0jabbYcaSiabbccaGiabb2gaTjabbwgaLjabbggaHjabb6gaUbqabaaaaaaakiaaxMaacaWLjaWaaeWaceaacqaIXaqmaiaawIcacaGLPaaaaaa@7848@

To calculate the relative fold-change in target gene expression, the MNE of the experimental sample was divided by the MNE of the control (Equation 2).

Relative fold-change = MNE_exp_/MNE_con _      (2)

### Cloning of the 5'-flanking promoter sequence by genome walking

A 3.4-kb 5'-flanking sequence of the *omTERT *gene was obtained using the Genome Walker kit (BD Biosciences) according to the manufacturer's instructions. Briefly, four genomic libraries were obtained by digestion of *O. melastigma *DNA with *Dra*II, *Eco*RV, *Pvu*II and *Stu*I restriction enzymes and subsequent ligation with an adaptor to the ends of the genomic fragments. Nested PCR amplifications were performed using two adaptor-specific primers provided in the kit, GW-AP1 (outer) and GW-AP2 (inner), in conjunction with two antisense GSPs, omTERT-5GW-GSP1 (5'-ACTGTCGGAAGGTTGGAGCAGAGTGG-3'; outer) and omTERT-5GW-GSP2 (5'-AGCGTCTGCGTGCGTTTGTACAGCGAC-3'; inner). A 50-μL PCR reaction contained 5 μL 10 × PCR buffer, 1 μL dNTP (10 mM), 1 μL GSP (10 μM), 1 μL adaptor primer (10 μM), 5 μL 1:50 diluted adaptor-ligated genomic library (for primary PCR) or 1:50 diluted primary PCR product (for secondary PCR) and 1 μL 50 × BD Advantage 2 polymerase mix (BD Biosciences). The amplification profile used was the same as that in RACE-PCR.

### Plasmid constructs

The *omTERT *5'-flanking region (nt. +48/- 3428; the transcription start site is defined as +1) was PCR amplified using primers with built-in restriction sites, TERTp-lucF (5'- AGACGCGTGGGGAAATTTGGAGTAACTG-3'; *Mlu*I site is underlined) and TERTp-lucR (5'-AA CTCGAGCAGGCGACAACTCAAGTGAC-3'; *Xho*I site is underlined). The PCR fragment (ca. 3.5-kb) was double digested with *Mlu*I/*Xho*I and subcloned into the pGL3-basic luciferase reporter vector (Promega) to produce pTERT-luc. The pCMV-omHIF-1α expression vector was generated by RT-PCR cloning of the coding region of *omHIF-1α *(DQ317443) using primers omHIF1α-F (5'-AACTCGAGCCTGACATGGACACAGGATTCGTACC-3'; *Xho*I site is underlined) and omHIF1a-R (5'-GTACGCGTTAACTGACTCAGATGACGTGGTCCAACG-3'; *Mlu*I site is underlined). The 2.2-kb RT-PCR product was subcloned into the *Xho*I/*Mlu*I site of the pCMV-TNT expression vector (Promega). All constructs were verified by automated DNA sequencing.

### Cell transfection and luciferase reporter assay

Materials for cell culture and transfection were obtained from Invitrogen, unless specified otherwise. The HIF-1α-deficient Chinese hamster ovary cell line (CHO Ka13) was a gift from Professor Peter Ratcliffe (Wellcome Trust Center for Human Genetics, Oxford University, UK). Cells were cultured in DMEM supplemented with 10% fetal calf serum, 1% antibiotics (penicillin G, 100 U/mL; streptomycin, 100 μg/mL) and 0.1 mM non-essential amino acids, in humidified air containing 5% CO_2 _at 37°C. CHO Ka13 cells seeded in 24-well plates at 1.1 × 10^5 ^cells per well were transfected with 600 ng of pTERT-luc or pGL3-basic reporter vector, 100 ng pCMV-omHIF-1α or pCMVTNT expression vector and 100 ng pTracer-EF/Bsd/*lacZ *(β-galactosidase expression vector) in LipofectAMINE 2000 and Opti-MEM reduced serum medium. After 18 h, cells were harvested with Glo-lysis buffer (Promega). Luciferase and β-galactosidase activities were measured using a Bright-Glo Luciferase Assay System (Promega) and a Beta-Glo Assay System (Promega), respectively. Interwell variations in transfection efficiency were corrected by normalization to β-galactosidase activity. Data given are mean ± SD from three independent experiments with duplicates in each experiment.

### *In situ *hybridization

*In situ *hybridization was performed to compare the cellular expression patterns of *omTERT *in testes and liver of normoxic and hypoxic fish. Briefly, whole fish was fixed in a cocktail of fixatives, dehydrated in a graded methanol series, infiltrated and embedded in paraffin. Serial sagittal sections (5-μm) were cut and mounted for *in situ *hybridization (for details, see [45]). A 300-bp *omTERT *fragment spanning motif E and motif E-I (nt. 2749–3048; Fig. 1) was cloned into the pGEM-T Easy vector (Promega), and digoxigenin (DIG)-labeled antisense and sense riboprobes were synthesized by *in vitro *transcription. Hybridization was performed overnight at 50°C with DIG-labelled riboprobes (0.5 ng/μl) in 2 × SSC, 50% formamide, 10% dextran sulfate, 50 μg/mL yeast tRNA and 5 U/mL RNase inhibitor. After hybridization, slides were washed in a graded series of SSC-0.1% Tween 20 at room temperature and incubated with anti-DIG antibody coupled to alkaline phosphatase (at 1:100 dilution; Roche Applied Science). Signals were detected using the NBT/BCIP substrate (Zymed). Sections were counterstained with Nuclear Fast Red and then examined by light microscopy (Carl Zeiss, Axioplan 2).

### Statistical analysis

Test for data homogeneity was conducted using Bartlett's test. Two-way analysis of variance (ANOVA) was used to test the null hypothesis that hypoxia does not cause significant changes in *omTERT*/*omHIF-1α*/*omEpo *gene expressions in testis/liver during the exposure period, and that no temporal changes occur in various gene expressions in testis/liver during the exposure period. Where significant differences were identified (*p *< 0.05) in treatment or in time, pairwise comparisons between the normoxia and hypoxia groups for the same time point, or pairwise comparisons between sampling intervals for the normoxia/hypoxia groups in testis/liver were carried out using Tukey's test. Likewise, Student's *t*-test was used to test that there was no significant difference in luciferase activities of pTERT-luc transfected cells in the absence/presence of omHIF-1α overexpression. Differences with *p *< 0.05 were considered significant. Statistical analysis was performed using the SigmaStat 3.0.1 package (SYSTAT Software Inc.).

## Authors' contributions

RMKY conducted most of the experimental work and data analyses in this study and drafted the manuscript. EXHC and PKSN performed the *in situ *hybridization and cell transfection experiments, respectively. HOLM assisted with the expression studies and data analysis. DWTA and RYCK initiated the project idea and contributed to the experimental design and editing of the manuscript.

## Figures and Tables

**Table 1:** Sequence identity of conserved motifs of omTERT to different vertebrate TERT proteins

Motif	Overall cross-species identity (%)	Percent sequence identity of omTERT and:					
		
		gTERT	fTERT	xTERT	cTERT	hTERT	mTERT
**N-terminal region**							
Region v-I	10.2	54.3	52.6	28.0	23.8	27.9	29.0
Region v-II	8.3	71.9	62.5	22.5	27.8	25.0	23.1
Region v-III	25.7	68.6	62.9	42.9	37.1	45.7	37.1
Region v-IV	25.0	75.8	69.5	43.3	42.7	41.9	42.7

**Central region**							
T motif	33.3	70.8	77.1	39.6	37.5	39.6	43.8
RT motifs:							
Motif 1	28.0	77.3	77.3	35.7	39.3	40.0	31.0
Motif 2	6.1	54.5	63.6	15.6	15.8	22.9	21.9
Motif A	29.4	82.4	70.6	50.0	47.1	50.0	41.2
Motif B'	47.1	94.1	82.4	52.9	58.8	64.7	58.8
Motif C	58.8	88.2	82.4	76.5	70.6	70.6	70.6
Motif D	34.6	76.9	80.8	57.7	53.8	57.7	53.8
Motif E	63.6	100.0	90.9	90.9	66.7	90.9	100.0

**C-terminal region**							
Region v-V	38.1	100.0	90.5	66.7	61.9	61.9	52.4
Region v-VI	47.2	83.3	79.2	54.2	54.2	58.3	56.9
Region v-VII	11.8	64.9	63.4	25.3	24.7	28.0	28.4

**Table 2:** Primers used in real-time PCR

Gene	Sense primer	Antisense primer	Amplicon size (bp)	Genbank accession number
*omTERT*	5'-CTGATGTGGAAGATGAAGGTGA-3'	5'-AGAAGCCAGGTCAGAAACAGAC-3'	122	DQ286654
*omEPO*	5'-GACCGGCTTGTGGCTGTT-3'	5'-CGTGTTGGTGACCGATGGT-3'	61	DQ415657
*omHIF1α*	5'-TGTGATATTTGCAGCATTCCA-3'	5'-CTACGACAGGAAATCCCATGA-3'	115	DQ317443
18S rRNA	5'-CCTGCGGCTTAATTTGACCC-3'	5'-GACAAATCGCTCCACCAACT-3'	134	DQ105650
